# The Bipolar Lithium Imaging Scan Study (BLISS): protocol for a 7T lithium-7 magnetic resonance study in bipolar disorder

**DOI:** 10.1093/psyrad/kkaf003

**Published:** 2025-03-07

**Authors:** Elvira Boere, Nic J A van der Wee, Albert M van Hemert, Andrew G Webb, Max de Leeuw

**Affiliations:** Leiden University Medical Center, Department of Psychiatry, Leiden, ZA 2333, The Netherlands; PsyQ ParnassiaGroup Mental Health Institute, Bipolar Disorder Outpatient Clinic, Rotterdam, MA 3062, The Netherlands; Leiden University Medical Center, Department of Psychiatry, Leiden, ZA 2333, The Netherlands; Leiden University Medical Center, Department of Psychiatry, Leiden, ZA 2333, The Netherlands; Leiden University Medical Center, Department of Radiology, Leiden, ZA 2333, The Netherlands; Leiden University Medical Center, Department of Psychiatry, Leiden, ZA 2333, The Netherlands; Rivierduinen Mental Health Institute, Bipolar Disorder Outpatient Clinic, Leiden, ZZ 2333, The Netherlands

**Keywords:** 7 Tesla, lithium, bipolar disorder, translational, ultra-high field, chemical shift imaging, clinical treatment response, lithium-7 MR imaging

## Abstract

**Introduction:**

Lithium treatment is considered the first-line option in the pharmacological treatment of bipolar disorder. At the same time, individual responses vary greatly, which complicates achieving rapid stabilization in many subjects with bipolar disorder. The neurobiological mechanism of action of lithium remains largely unknown, hindering the development of clinically applicable predictors of individual treatment responses. The recent introduction of ultra-high-field lithium magnetic resonance imaging (MRI) has opened up a promising avenue for better linking brain measures with clinical response to lithium treatment.

**Methods and analysis:**

This is an observational study involving 80 adults with bipolar disorder who begin lithium as part of their regular treatment. Within 4 weeks of reaching stable therapeutic serum lithium concentrations, brain lithium concentrations will be measured by employing a 3D lithium-7 chemical shift imaging (^7^Li CSI) sequence on a 7T MR system. The primary outcome is the clinical response to lithium treatment at 1 year follow-up, assessed using a validated questionnaire. Linear regression analysis will be used to establish correlations between brain lithium concentrations—measured through mean brain, voxel-wise, parcellation, and region-of-interest approaches—and clinical lithium response.

**Ethics and dissemination:**

The BLISS study protocol (NL80214.058.22) has been approved by the Medical Ethics Committee of Leiden, The Hague, and Delft in The Netherlands. Results will be submitted for publication in peer-reviewed journals and shared with the key population.

Registration Online at clinicaltrials.gov (NCT06134349), 20 November 2023.

## Introduction

Bipolar disorder (BD) is a chronic affective and incapacitating disorder. Around 50% of the time subjects with BD experience depressive or manic symptoms (Kupka *et al*., 2007), resulting in severely impaired health-related quality of life and high societal costs (Bessonova *et al*., 2020).

Pharmacotherapy forms the backbone of psychiatric care for BD, with lithium being the first treatment option, as studies have consistently demonstrated its stabilizing properties (Bauer *et al*., 2003; Kessing *et al*., 2018; Miura *et al*., 2014; Severus *et al*., 2014).

However, despite lithium treatment typically being guided by serum lithium concentrations with reference values based on pharmacokinetic studies (Nolen *et al*., 2019), individual responses to lithium tend to vary greatly. More specifically, multiple studies have reported that only 30–33% of subjects achieve full remission of BD following lithium treatment, with a similar percentage reaching partial remission, and the remainder showing no signs of remission (Baldessarini *et al*., 2000; Garnham *et al*., 2007; Maj *et al*., 1998; Rybakowski *et al*., 2001).

In addition, individual lithium responses in BD subjects can only be evaluated after 12 weeks of treatment, and some studies even suggest waiting up to 6 months (Tohen *et al*., 2009). This delay may be linked to the strong, slow, and indirect effects of lithium on several key neurotransmitter systems within relevant brain circuitry (Alda, 2015). This has multiple clinical and societal implications, as it hinders the rapid stabilization of BD.

In recent decades, several biological correlates of lithium treatment have been identified. Amongst the most replicated findings are the inhibition of glycogen synthase kinase 3β, a decrease of inositol, regulation of intracellular calcium homeostasis, and attenuation of glutamatergic activity following lithium treatment, demonstrating a shift away from biological profiles that are found to be aberrant in BD towards profiles more similar to those of healthy controls (Alda, 2015; Kato, 2019). However, despite these findings, a robust biomarker for clinical lithium response is still lacking, and there remains an unmet need for a better biological conceptualization of lithium treatment response (Bauer *et al*., 2018).

A promising approach to address this need has emerged with the recent introduction of ultra-high-field 7T magnetic resonance (MR) systems, which enable the assessment of brain lithium concentrations *in vivo* at higher resolution.

Magnetic resonance imaging (MRI) and spectroscopy (MRS) are methods to non-invasively measure the distribution of lithium in the brain. The main challenges are the very low signal-to-noise ratios (SNRs) compared to conventional ^1^H images due to the much lower ^7^Li concentration (<1 mM compared to 110 M for water) (Soares *et al*., 2000). ^7^Li MRS studies at 1.5T were among the first to demonstrate that given a serum lithium concentration, brain lithium concentrations between subjects would vary greatly (Sachs *et al*., 1995). However, these measurements were acquired from a large section of the brain (a 6 cm axial slab), providing no information on spatial distribution, and took 21 min to acquire. Following the introduction of 3T and 4T MRI, the improved SNR allowed for some degree of estimation of the ^7^Li spatial distribution. At 3T, three-dimensional (3D) images were acquired with 25 mm isotropic spatial resolution (15.6 ml) in 8 min (Smith *et al*., 2018). MRS imaging results at 4T were acquired in 46 min with a spatial resolution of ∼12 ml per voxel (Lee *et al*., 2012).

To date, only one paper has reported on ultra-high-field ^7^Li MRI in lithium-treated BD subjects (Stout *et al*., 2020). This study localized the lithium signal to seven large brain regions of interest (brainstem, cerebellum, frontal lobe, midbrain, occipital lobe, parietal lobe, and temporal lobe), identifying consistently high lithium concentrations in the left hippocampus and right pallidum cross-sectionally in a cohort of 21 BD subjects.

Kato *et al*. (1994) demonstrated at 1.5T that brain lithium concentrations, and not serum lithium concentrations, correlated with clinical improvement following 4 weeks of lithium treatment in BD subjects with a manic episode. Machado-Vieira *et al*. (2016) demonstrated that brain lithium concentrations correlated with serum lithium concentrations only in BD subjects who had reached clinical remission following 6 weeks of lithium treatment in BD. In this sample, aged 18–45 years, the authors also found that brain lithium concentrations inversely correlated with age. This inverse correlation could not be replicated in another ^7^Li MRS study at 1.5T in adults (mean age 37 ± 9 years) and children with BD, although a higher brain-to-serum ratio did correlate with increasing age (Moore *et al*., 2002). Clinical lithium response was not reported in the latter paper.

A heterogeneous lithium distribution pattern, suggesting that lithium may target specific brain regions, was found in a study at 3T that included eight euthymic BD subjects, one of whom was classified as a full responder (Smith *et al*., 2018). Brain lithium concentrations did not correlate with serum lithium concentrations in this study.

A 4T study that included 15 lithium-treated BD subjects, all with a depressed or mixed mood episode at the time of scan acquisition, also found a heterogeneous lithium distribution. Here, brain lithium concentrations correlated strongly with serum lithium concentrations, but clinical response was not reported (Lee *et al*., 2012).

Interestingly, the findings of Kato *et al*. (1994) and Machado-Vieira *et al*. (2016) suggest that brain lithium concentrations correlate with clinical response to lithium treatment. Intracerebral homeostasis of ions, such as lithium, is mainly modulated by (i) the blood–brain barrier (BBB), which consists of endothelial cells that provide a barrier function between peripheral and cerebral blood circulation (Benz and Liebner, 2022), and (ii) the blood–cerebrospinal fluid barrier (BCSFB), which mainly consists of choroid plexus secretory epithelial cells (Spector *et al*., 2015).

Regarding intracerebral lithium ion homeostasis specifically, recent preclinical findings suggest that lithium is actively transported through the BBB, and that this transport is mediated by several types of sodium-related transporters and channels, which are coded for by members of the solute carrier gene family (Luo *et al*., 2021). Moreover, genetic polymorphisms and lithium itself have been found to modulate the expression of these genes, potentially providing a key mechanism of action in the intracerebral availability of lithium.

The BCSFB has been studied to a lesser extent; however, a predominant passive paracellular diffusion process is suggested to underlie lithium transport here, as the BCSFB shows greater permeability than the BBB (Luo *et al*., 2021).

Taken together, preclinical findings suggest that brain lithium concentrations may be predominantly attributed to the lithium-transporting properties of the BBB.

Additionally, Smith *et al*. (2018), Lee *et al*. (2012), and Stout *et al*. (2020) reported an uneven distribution of lithium throughout the brain. This uneven distribution pattern is probably indicative of regional variations in lithium uptake, which may be influenced by the spatial distribution of genetically expressed solute carrier genes.

The relevance of this uneven distribution regarding the mechanism of action of lithium and clinical treatment response has yet to be established.

The current Bipolar Lithium Imaging Scan Study (BLISS) aims to establish correlations between baseline brain lithium concentrations in subjects who have reached stable therapeutic serum lithium concentrations within 4 weeks prior to scan acquisition and (i) longitudinal clinical lithium treatment outcome measures, and (ii) serum lithium concentrations.

For clarification, it is important to note that the current 7T BLISS study is not related to a previous study with the same acronym, nor to the R-LiNK consortium, both of which focused on 3T lithium imaging and spectroscopy in subjects with BD (Smith *et al*., 2018; Scott *et al*., 2019; Little *et al*., 2024).

## Methods

### Design

**Figure 1:**
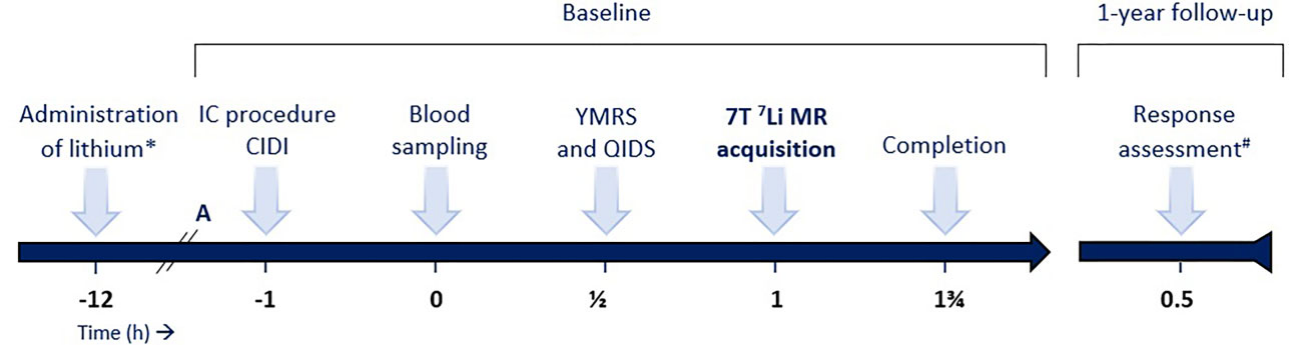
Overview of the Bipolar Lithium Imaging Scan Study. * Part of standard care; A = Arrival at the hospital; IC = Informed Consent; CIDI = Composite International Diagnostic Interview; YMRS = Young Mania Rating Scale; QIDS = Quick Inventory of Depressive Symptoms; # = Retrospective Criteria of Long-Term Treatment Response in Research Subjects with Bipolar Disorder scale.

This is an observational study of adult subjects with BD who have started lithium treatment as part of standard care. A detailed, graphical overview of the study is provided in Fig. 1.

### Study population

The following inclusion criteria will be applied: (i) age 18 years or older, (ii) a clinical diagnosis of BD type I or type II (Kessler *et al*., 2013), (iii) having reached stable therapeutic serum lithium concentrations (reference values 0.6–1.0 mM in those aged < 65 years; 0.4–0.8 mM in those aged ≥ 65 years) within 4 weeks prior to study participation, and (iv) provided written informed consent.

The following exclusion criteria will be applied: (i) insufficient comprehension of the Dutch language, (ii) inability to provide informed consent, (iii) drug or alcohol misuse within 2 weeks prior to study participation, and (iv) meeting any exclusion criterion for MRI.

### Study procedures

Baseline assessments take place at the Leiden University Medical Centre, after obtaining written informed consent and within 4 weeks of reaching a stable therapeutic serum lithium concentration. A blood sample will be collected 12 h (± 30 min) after the last lithium intake to determine the serum lithium concentration. Quantitative analysis of lithium in serum will be performed using a Multigent Lithium Assay (Sentinel Diagnostics, Italy): this colorimetric assay relies on a chromogenic chelator that selectively binds lithium ions at an alkaline pH. Two questionnaires assessing manic (Young *et al*., 1978) and depressive (Rush *et al*., 2003) symptom severity will be carried out to assess mood state during scan acquisition.

At 12 months of follow-up, the longitudinal outcome of lithium treatment will be assessed using a validated questionnaire (Manchia *et al*., 2013), via a telephone interview.

### Scanner and image acquisition

At the Gorter Center of the Department of Radiology at Leiden University Medical Centre in The Netherlands, imaging data will be acquired on a 7T MR system (Achieva, Philips Medical Systems, Eindhoven, The Netherlands) using a dual-tuned ^1^H/^7^Li volume head-coil (RAPID Biomedical GmbH, Rimpar, Germany). Datasets for each participant are created utilizing a 3D chemical shift imaging sequence (^7^Li CSI) [TR = 1000 ms, TE 1.66 ms, FOV = 240 (feet/head) × 240 (anterior/posterior) × 220 (right/left) mm, matrix size = 12 × 12 × 11, bandwidth 512 Hz, 64 complex data points acquired, 2 averages (Hanning acquisition weighting), voxel size = 20 × 20 × 20 mm, nominal spatial resolution = 8000 mm^3^].

Anatomical scans will be acquired as T1-weighted fast gradient echo sequences using an inversion time of 1000 ms. These will be used solely as an overlay to help accurately localize the lithium signal in 3D space. Anatomical and lithium images will be inherently aligned since they are acquired with a double-tuned head coil, assuming no head movement between scans, which is ensured by head stabilization with foam cushions. Additionally, the large spatial resolution of the lithium images reduces the likelihood of misalignment. Matlab will be used to set up a pipeline to register the ^7^Li CSI images with the anatomical scans. Overlays will be created using FMRIB Software Library (FSL). The lithium images will be interpolated; however, this does not affect their intrinsic spatial resolution. Total scan duration is ∼30 min.

### Lithium concentration quantification

To estimate lithium concentration from the intensity of the images, we have adapted our methods from the only protocol to date that used essentially the same hardware set-up (Stout *et al*., 2017, 2020). Based on this protocol, images were acquired from two uniformly filled 7 litre cylindrical phantoms of lithium carbonate (1 and 10 mM, respectively, pH 7.0) at room temperature; T1 and T2 relaxation times of lithium were lowered by adding copper sulphate (1.2 g/l). 3D datasets were acquired using a 3D phase-encoding CSI sequence with spatial resolution of 20 × 20 × 20 mm with a data acquisition time of ∼25 min.

3D ^7^Li CSI scans were used to minimize the effects of the very short T2* of ^7^Li in the brain. Stout *et al*. (2020) report the T2 value of brain lithium to be 63 ms at 7T, which would represent the T2* value with perfect shimming across the entire brain. Based on initial *in vivo* data using a 3D iterative shimming approach, we estimate the T2* value to be less than 10 ms based on the spectral linewidth.

The theoretical SNR of this 3D ^7^Li CSI sequence is comparable to that of fast imaging sequences used in previous studies, including non-Cartesian sampling ultrashort echo-time sequences (Stout *et al*., 2020) and Cartesian balanced steady-state free precession (Smith *et al*., 2018), each with distinct SNR characteristics. Pure phase encoding intrinsically has ∼10% penalty in SNR per dimension compared to frequency encoding if the effects of T2/T2* relaxation are ignored. Therefore, a 3D phase-encoded sequence may have a slight penalty of a few per cent compared to a 2D phase, 1D frequency-encoded sequence. Ultra-short echo-time sequences are useful for very short T2* values, but with a T2* of a few milliseconds, the TE time of 1.66 ms in the 3D CSI sequence results in relatively low signal loss. While balanced steady-state free precession (SSFP) sequences conventionally provide the highest SNR per unit of the square root of time, this may not necessarily be true for lithium, as its SNR depends on the T2/T1 ratio, with lithium having a very long T1 and relatively short T2.

Under the assumption that the SNR increases as the 3/2 power of the strength of the main magnetic field (B0), the SNR at 7T compared to 3T should be ~3.5 times higher. This means that at 7T, the linear dimensions of a voxel should be ~ 70% of those at 3T to give an equivalent SNR.

**Figure 2:**
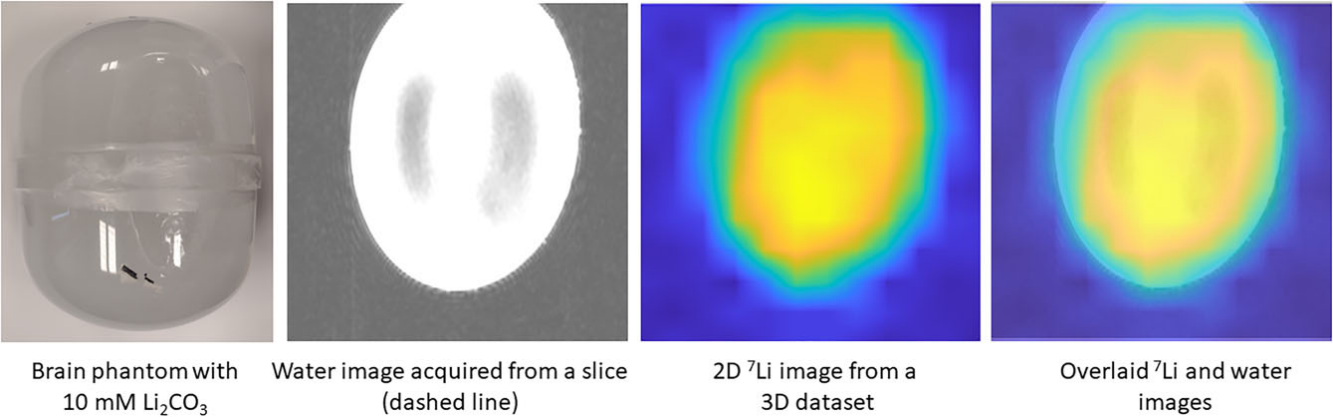
Lithium carbonate 10 mM phantom map.

**Figure 3:**
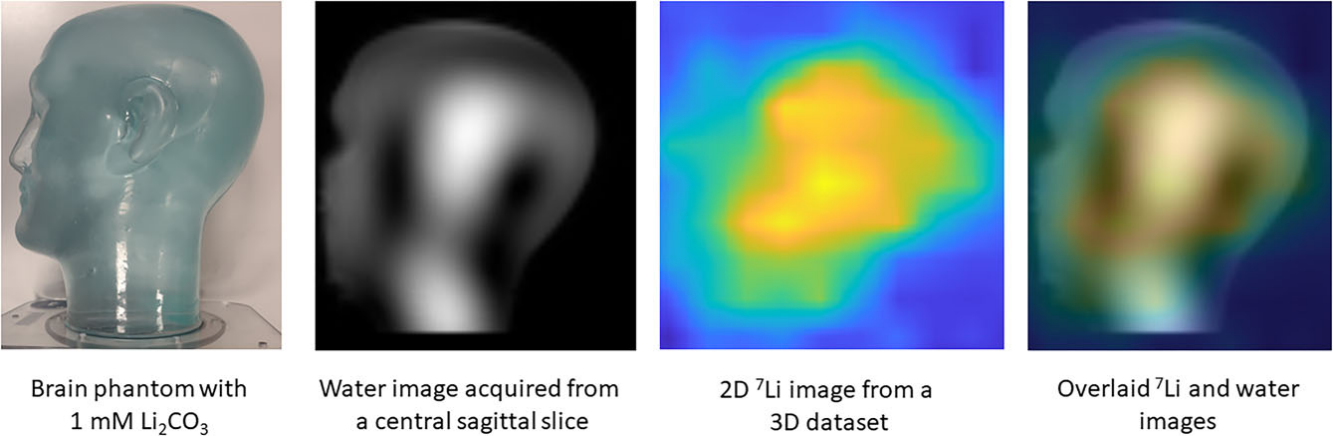
Lithium carbonate 1 mM phantom map.

**Figure 4:**
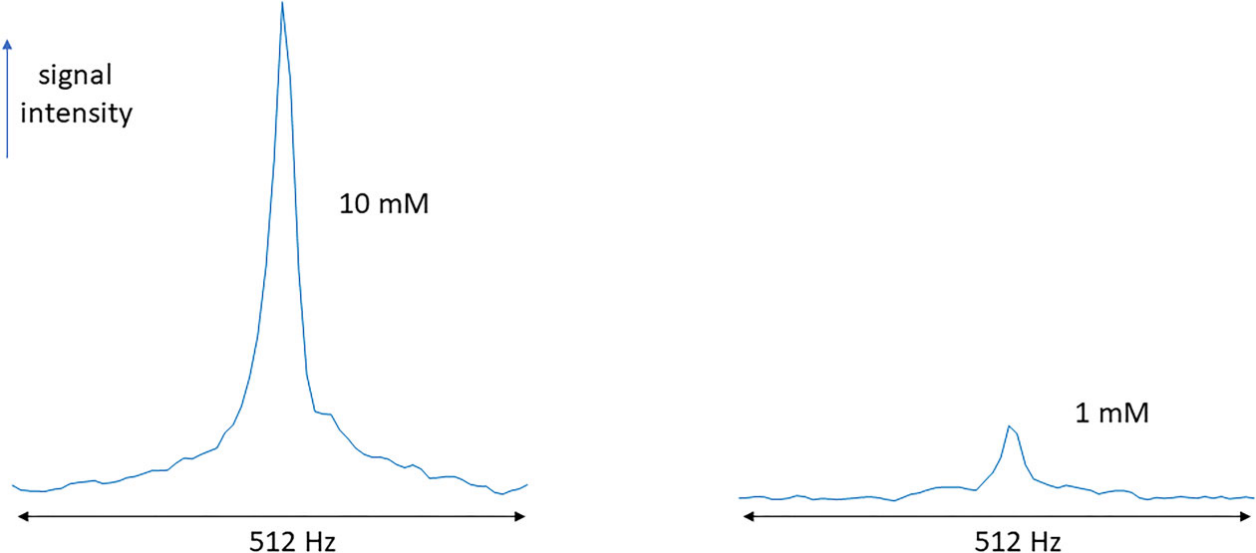
Sample spectra from the 7T ^7^Li CSI sequence showing the expected quality at 10 and 1 mM.

Maps were produced by integrating the area under the lithium peak for each spatially resolved spectrum (Figs 2–4). The signal non-uniformities in the proton images arise from the wavelength of the radiofrequency at 7T in water being smaller than the dimensions of the phantom, leading to wave interference effects. As lithium carbonate is insoluble in oil, acetone, or other low-permittivity liquids, it was not possible to use a phantom with lower relative permittivity and thus a longer wavelength. As a result, these particular images are primarily useful for outlining the outer edges of the phantom for anatomical overlay. The lithium signal also appears inhomogeneous, with lower intensity near the edges of the phantom. While there is a small contribution from the dielectric effect, the primary cause is the relatively broad point spread function (PSF), which can be interpreted as a sinc function modulated by the pseudo-Hanning acquisition weighting applied in the data acquisition. The intrinsic digital spatial resolution is 20 mm, given by the field-of-view divided by the number of data points acquired in each phase-encoding dimension. Since we apply a Hanning acquisition weighting to reduce the lobes of the otherwise sinc-shaped PSF, this broadens the central peak of the PSF, increasing the full-width-half-maximum to between 25 and 30 mm in each phase-encoded direction.

For lithium data analysis, each lithium-free induction decay will be multiplied by a matched exponential function. 3D inverse Fourier transformation will be performed with respect to each phase-encoding direction, followed by forward Fourier transformation in the time domain. The resulting spectra will be integrated to give a single value for each of the 12 × 12 × 11 voxels, corresponding to the localized ^7^Li signal in that voxel. Creating overlays of the 7T ^7^Li CSI data sets with anatomical scans helps identify areas affected by partial volume effects, thereby potentially reducing the risk of misinterpretation.

Individual brain lithium concentrations will be assessed using mean brain, voxel-wise, parcellation, and region-of-interest approaches. Parcellation involves dividing the brain into regions (parcels) for network analysis, to identify meaningful divisions that reflect the brain's organization regarding functional lithium uptake. Region-of-interest analysis, on the other hand, targets specific predefined areas: according to the findings of a recent systematic review on the effects of lithium on frontolimbic circuitry, these areas include the hippocampus, amygdala, prefrontal cortex, and anterior cingulate cortex (Boere *et al*., unpubl. data). We have decided to adopt these multiple approaches, as it is not yet clear which approach will be the most relevant.

### Outcome measures

#### Main study parameter

Clinical lithium response will be assessed using the Retrospective Criteria of Long-term Treatment Response in Research Subjects with Bipolar Disorder scale, which has been validated for research purposes (Manchia *et al*., 2013). This scale quantifies BD symptom improvement over the course of treatment (A score; range 0–10), which is then weighted against five criteria (B score) that assess confounding factors, each scored 0, 1, or 2. The total score is calculated by subtracting the total B score from the A score, with a range from 0, indicating lithium non-response, to 10, indicating excellent lithium response.

#### Secondary study parameter

Serum lithium concentrations will be determined to establish correlations with brain lithium concentrations.

#### Other study parameters

The Young Mania Rating Scale is a validated clinician-administered rating scale to assess manic symptom severity (Young *et al*., 1978). The Quick Inventory of Depressive Symptoms is a validated depression rating scale to assess depressive symptom severity (Rush *et al*., 2003). Both measures will be used to assess mood state on the day of scan acquisition.

## Data handling and analysis

The handling of data will be in line with the EU General Data Protection Regulation and the Dutch Act on Implementation of the General Data Protection Regulation. Data will be electronically stored in Castor EDC, except imaging data.

Each study subject will receive a personal identification number and data will be stored pseudonymously. The data dictionary will be exported every time the electronic Case Report Form is updated. Access to Castor EDC will only be provided to authorized staff.

After completion of inclusion and follow-up assessments, the Castor EDC database will be locked. Data will be kept in storage for 15 years.

Imaging data will be acquired and stored under each participant's identification number, on a secure internal network that is only accessible to authorized staff.

Serum lithium concentrations will be determined on the same day on which blood is drawn, and samples will be destroyed directly thereafter.

### Statistical analysis

#### Sample size calculation

A straightforward power analysis was not possible due to the newly developed 7T ^7^Li CSI acquisition method. However, in a broader context, as the effect size under the alternative hypothesis is typically unknown, it is common practice in MR studies to estimate the number of participants based on previous empirical experience in the field and the assumptions from imaging analysis software.

Following this, we based our sample size calculation on the only relevant paper to date that has studied brain lithium concentrations in lithium-treated BD subjects using essentially the same hardware set-up (Stout *et al*., 2020). Their study identified a cluster of high lithium concentrations across 21 study subjects. The sample was characterized by a mean brain lithium concentration of 0.33 mmol/l (SD 0.06) and a mean score on the clinical treatment response scale of 6.1 (SD 2.5) (Manchia *et al*., 2013). However, given that the latter scale has a range of 0–10 and the raw data were unavailable, it was not possible to cluster the scores with the extent of clinical lithium response. Additionally, due to the design of the current BLISS protocol, long-term lithium treatment response will be unknown at baseline.

Following these considerations, we estimate that 21 subjects will be needed in the group of full lithium responders to be able to discriminate between lithium responders and lithium non-responders. Since only around 33% of study participants are expected to qualify as full lithium responders (Baldessarini et al., 2000; Garnham *et al*., 2007; Maj *et al*., 1998; Rybakowski *et al*., 2001), and response status is not yet known during scan acquisition, we estimate that 63 participants will be required for inclusion. Additionally, based on dropout rates estimated at 25% from other studies (Stange *et al*., 2018; Clausen *et al*., 2019), we anticipate a final sample size of 80.

#### Data analysis

##### Primary study parameters

Linear regression analysis will be used to establish correlations, with lithium concentrations in the brain, derived from various approaches, as independent variables, and continuous scores on the Retrospective Criteria of Long-term Treatment Response in Research Subjects with Bipolar Disorder scale as the dependent variable.

##### Secondary study parameters

Linear regression analysis will be used to establish correlations between lithium concentrations in the brain and in the serum.

##### Other study parameters

Scores on the rating scales for manic and depressive symptoms during scan acquisition will be used for exploratory analysis. Subgroup analyses by mood state will be performed if the assumptions are met.

## Ethics and dissemination

The BLISS study protocol (NL80214.058.22) has been approved by the Medical Ethics Committee of Leiden, The Hague, and Delft in accordance with Article 82 of the European Medical Device Regulation 2017/745. The study will be conducted following the Declaration of Helsinki, Good Clinical Practice guidelines, and the Dutch Medical Research Involving Human Subjects Act. Written informed consent will be obtained from all participants prior to study participation.

The BLISS study is subject to on-site monitoring, and the investigator will provide the Ethics Committee with an annual progress report on the study.

The results of this study will be submitted for publication in peer-reviewed journals and shared with the key population.

## Safety and safety monitoring

The ultra-high-field 7T MR system is widely used in research settings, and since its introduction in the 1990s, no serious adverse events have been reported. Important temporary side effects include vertigo, nausea, and involuntary eye movements, due to forces on ion currents in the semicircular canals. All individuals entering the 7T MR system are provided with adequate sound protection to reduce acoustic noise, thereby protecting the ears and enhancing comfort during scan acquisition. Slightly modified scanner software will be used without compromising safety (van Osch and Webb, 2014).

Regarding safety in lithium-treated BD subjects, two previous studies using conventional ultra-high-field MRI in similar populations to those in the current BLISS study reported no safety issues (Athey *et al*., 2021; Rootes-Murdy *et al*., 2019). The only other study to date combining a ^1^H/^7^Li volume head-coil with ultra-high-field MRI also reported no safety issues (Stout *et al*., 2020). Based on these findings, no additional risks are expected in ultra-high-field ^7^Li MRI.

Given the estimated low-risk profile of the current study, regular monitoring of (serious) adverse events was deemed sufficient, and no additional safety board was installed.

## Discussion

To our knowledge, the current BLISS protocol is the first study worldwide to establish correlations between highly specialized 7T ^7^Li CSI data in BD subjects and longitudinal measures of clinical lithium response. The main strengths of the current protocol are: (i) this acquisition method is expected to provide novel insights into the direct mechanisms underlying clinical lithium response, and (ii) the participation of real-world BD subjects greatly enhances the future generalizability of our findings.

At the same time, there are potential pitfalls to address. For instance, severe manic or depressive mood states may hinder recruitment and assessments, potentially leading to selection bias. Also, the 12-month interval between assessments may increase the risk of loss to follow-up, which has been accounted for in the sample size calculation. Lastly, ultra-high-field MRI may currently be considered expensive. However, broader future implementation of this technique is expected to lower scan costs. Ultimately, the BLISS study aims to contribute to more rapid stabilization of bipolar disorder, which, in turn, is expected to reduce the substantial personal and societal costs associated with this condition (Bessonova *et al*., 2020).

If the BLISS study demonstrates that brain lithium concentrations within 4 weeks of reaching stable serum lithium concentrations predict clinical lithium response at 1 year of follow-up, it is expected to provide key data to a future neuroimaging phenotype of lithium treatment response in BD. Such a phenotype would contribute to personalized medicine by enabling the identification of likely responders and non-responders at an earlier stage of treatment. In particular, the early identification of BD subjects likely to respond to lithium treatment may help overcome doubts or improve adherence, while identifying those unlikely to respond could provide BD subjects and their clinicians with the guidance to switch to alternative treatments at an earlier stage. In this way, both responder groups are expected to benefit from accelerated clinical stabilization.

## Study status

Recruitment began in March 2024, and completion of inclusions is expected by March 2027.
